# Effect of Midwifery Students’ Continuity of Care on Childbirth Fear and Postpartum Depression: An Individually Randomized Two-Arm Superiority Trial

**DOI:** 10.34172/hpp.45154

**Published:** 2026-08-21

**Authors:** Elham Jafari, Mojgan Mirghafourvand, Shamsi Abbasalizadeh, Shahla Meedya, Leila Doshmangir, Sakineh Mohammad-Alizadeh-Charandabi

**Affiliations:** ^1^Student Research Committee, Tabriz University of Medical Sciences, Tabriz, Iran; ^2^Social Determinants of Health Research Center, Tabriz University of Medical Sciences, Tabriz, Iran; ^3^Department of Midwifery, Faculty of Nursing and Midwifery, Tabriz University of Medical Sciences, Tabriz, Iran; ^4^Women’s Reproductive Health Research Center, Tabriz University of Medical Sciences, Tabriz, Iran; ^5^Centre for Nursing and Midwifery Research, School of Nursing and Midwifery, Western Sydney University, Sydney, Australia; ^6^Department of Health Policy and Management, Tabriz Health Services Management Research Center, School of Management and Medical Informatics, Tabriz University of Medical Sciences, Tabriz, Iran

**Keywords:** Continuity of patient care, Education, Medical, Undergraduate, Iran, Mental health, Midwifery, Pregnancy, Quality of health care, Students

## Abstract

**Introduction:**

Midwifery continuity of care may improve maternal mental health. Integrating this model into midwifery education may facilitate broader adoption; however, no trials examined its effects on maternal health. We aimed to assess the effect of a midwifery student’s continuity of care program on fear of childbirth and postpartum depression (primary outcomes).

**Methods:**

In this trial, low-risk women at 26–29 weeks of gestation were recruited from Tabriz health centers from November 2022 to February 2023 and assigned to intervention or control groups using stratified block randomization. Twenty-five final-year midwifery students provided continuity of care (each for one to two women) from pregnancy through postpartum for the intervention group; both groups received usual care. Childbirth fear and depression were assessed using the Wijma Delivery Expectancy/Experience Questionnaire and Edinburgh Postnatal Depression Scale at baseline, and 40–50 days postpartum. Analysis of covariance, adjusted for baseline values, parity, and intended hospital type, was used to compare the groups.

**Results:**

All 93 recruited women were analyzed; 50% were primiparous. In the intervention group (n=45), 76% attended≥6 antenatal sessions, and 64% participated in≥3 postpartum sessions provided by the students. The intervention group showed lower mean total fear of childbirth (35.8 vs. 75.8, adjusted mean difference (AMD) -43.2; 95% CI: -52.7 to -33.7) and depression scores (5.3 vs. 11.2, AMD -5.9; 95% CI: -8.1 to -3.7) than controls.

**Conclusion:**

The findings support further evaluation and implementation research. However, these findings should be interpreted cautiously considering the single-city design, lack of blinding, and reliance on self-reported outcomes.

**Trial registration:**

Iranian Registry of Clinical Trials, IRCT20100414003706N41, registered prospectively on 30 April 2022, https://www.irct.ir/search/result?query = IRCT20100414003706N41.

## Introduction

 Fear of childbirth (FOC) and postpartum depression (PPD) are major contributors to psychological birth trauma, affecting women’s mental health and well-being.^1^ A review reported a wide discrepancy in FOC prevalence, ranging from 0.7% to 89% across cultures and countries.^2^ In Iran, reported FOC prevalence ranges from 17% to 89%.^3^ The global PPD prevalence is 26.3%,^4^ with an estimated 28.7% in Iran.^5^ FOC is associated with increased caesarean section requests, preterm labor, prolonged labor, postpartum depression, and post-traumatic stress disorder.^6^ PPD is also linked to many adverse outcomes, such as poor psychological health, impaired maternal-infant bonding, relationship difficulties, sexual dysfunction, and reduced quality of life.^7^

 Midwifery-led continuity of care (MCOC) is a key framework for enhancing the psychological well-being of women with FOC.^8-10^ A 2024 overview and a 2025 systematic review found that prenatal education, psychoeducation, and counselling may reduce FOC.^11,12^ MCOC may also play a critical role in managing perinatal depression by fostering a trust-based relationship between women and midwives.^13-15^ However, a recent systematic review found limited evidence on the effect of midwifery-led caseload care on PPD.^16^

 Midwifery student continuity of care (MSCOC) is a specific form of MCOC in which students provide continuous care to childbearing women. It is part of the midwifery training requirements in some European countries.^17^ However, it has been implemented in only one low- and middle-income country, Indonesia.^18^ Qualitative and observational studies emphasize that women value emotionally supportive, meaningful relationships with students in MSCOC settings.^19-23^

 In Iran, midwives’ roles in low-risk pregnancy and childbirth are often overlooked^24^ due to the medicalization of maternity care.^25^ This medicalization has diminished midwifery authority and hindered a woman-centered approach.^26^ Despite initiatives to promote physiological birth, including childbirth preparation classes and guidelines,^27^ the caesarean section remains high at 52%.^28^ Fear of childbirth, alongside negative experiences, poor communication, and insufficient continuous care, is a key reason for requesting a caesarean.^29^ Limited evidence from Iran suggests that midwifery-led care improves vaginal birth rates and childbirth satisfaction.^30,31^ Also, a trial in Iran found that a mHealth application providing continuous support reduced FOC.^32^ To transition to MCOC, reforms in midwifery education, policy, and supportive environments are necessary.^17^ Strengthening education and empowering midwifery students in MCOCs are essential for improving service quality and achieving lasting improvements in Iran’s midwifery services.^17,33^

 Reviews highlight that 90% of MCOC studies have been conducted in high-income countries,^18^ emphasizing the need for research in low- and middle-income countries.^34^ We also found only limited observational studies and no randomized controlled trials on the effect of MSCOC on maternal health. Therefore, we aimed to implement the MSCOC program during pregnancy, labor, and the postpartum period and to examine its effects on fear of childbirth and postpartum depression.

## Methods

###  Study Design

 This study was a two-parallel-arm, superiority, individually randomized controlled trial (RCT). Due to the nature of the intervention, blinding the participants and care providers was not possible. Blinding the outcome assessor was also not possible, as outcomes were self-reported by the participants. This paper adheres fully to the CONSORT guidelines.

###  Participants

 The inclusion criteria for women were singleton pregnancy, gestational age between 26 to 29 weeks, at least six years of education, a history of up to two vaginal births without prior caesarean section, and willingness to have a vaginal birth at participating hospitals. Exclusion criteria included underlying diseases, high-risk pregnancy history, current pregnancy complications, recent stressful events, major fetal abnormalities, lack of smartphone or internet access, or participation in another trial.

 Initially, recruitment was limited to women intending to give birth at two teaching hospitals in Tabriz. Approximately two months after recruitment began, before any data analyses, due to slow enrolment progress, eligibility criteria were expanded to include women intending to deliver at all hospitals with high delivery volumes in the city. One hospital declined permission to participate, resulting in the final addition of one social security hospital and six private/semiprivate hospitals. The expansion was done after approval of the research council, but the registered protocol was not amended to reflect this change. To account for potential differences related to hospital setting, randomization was stratified by hospital type (teaching, social security, and private/semi-private hospitals).

 Twenty-five of 28 final-year midwifery students from Tabriz University of Medical Sciences, with written informed consent, participated as MSCOC providers in the intervention group.

 The principal investigator (PI, first author, PhD student in midwifery) mentored the students. She is an experienced midwifery instructor and a member of the physiological childbirth training core.

###  Recruitment 

 Women from all public health centres in Tabriz, Iran, with at least four potentially eligible pregnant women were recruited. The PI identified potentially eligible women through the national integrated health system (IHS) entitled “SIB”. She then contacted the women by phone, provided study details, and invited them to visit the health centre to participate in the trial. After further eligibility screening and providing detailed study information, written informed consent was obtained from eligible women. The women were then recruited into the study and completed the baseline questionnaires.

###  Sample Size

 The sample size was calculated using G*Power software based on an independent samples t-test (two-sided), considering the two pre-specified primary outcomes: postpartum depression (PPD) and fear of childbirth (FOC). A two-sided α of 0.05, 90% power, and a 1:1 allocation ratio were assumed.

 Based on a previous Iranian study^35^, assuming a mean PPD score of 14.9, a standard deviation of 4.0, and a clinically significant reduction of 2.9 points (20% reduction), 40 participants per group were required. After allowing for 15% possible attrition, the final sample size was set at 46 participants per group. For FOC, based on Andaroon et al.^36^, assuming mean W-DEQ scores of 63.9 and 51.1 in the control and intervention groups, respectively (common SD: 14.1), the required sample size was 33 participants per group. Therefore, the sample size determined based on PPD was considered the final sample size.

###  Sequence Generation and Randomization 

 The allocation sequence was generated using a computer program with randomly varied block sizes of 4 and 6 (1:1 ratio), stratified by parity (nulliparous, multiparous) and intended hospital type for delivery (teaching, social security, private/semiprivate). The sequence was generated by a person not involved in recruitment, randomization, intervention, or data collection. We used a central method to conceal allocation from the investigator (PI) who recruited and assigned participants into the groups. The variable block sizes were concealed from the investigator responsible for participant recruitment and allocation to prevent prediction of upcoming intervention assignments. After participant recruitment and receiving written informed consent, while individuals were completing the baseline questionnaires, the PI sent the participant’s name, mobile number, and stratification factors (i.e., parity and intended hospital type for delivery) to the sequence generator via text message, who informed the PI of the woman’s group based on the predetermined sequence after completion of baseline questionnaires.

 Simple randomization was used to assign women in the intervention group to midwifery students. Each student was responsible for one to two women as primary care providers, and supported one to two others assigned to another student. Supporters were selected based on their relationships with primary care providers, ideally with one being from Tabriz. On the recruitment day, the primary student care provier (and, if possible, her supporter) and the PI attended the centre, where participants in the intervention group were introduced to their student care providers. If the assigned student was inaccessible at the recruitment day, the PI contacted the next student on the randomization list. Pregnant women were assigned to inaccessible students according to a new randomization sequence.

###  Intervention

####  Student training and mentoring

 Before recruitment, students attended four 4-hour workshops covering communication skills, assertiveness, critical thinking, decision-making, physiological childbirth, evidence-based midwifery, the philosophy of continuity of care, and Iranian national maternal health guidelines. The mentor organized and taught the workshops.

 The mentor established a Telegram group for student communication throughout the study to enhance their learning and support. She shared educational content in PDFs with audio and brief videos, and also facilitated weekly online question-and-answer sessions to address students’ questions. Students also shared relevant cases related to mothers under their care, such as anonymous test results or audio files. The mentor facilitated discussions and provided comprehensive explanations. Additionally, the mentor privately sent step-by-step training content for each care session to students, two to three days before the scheduled session.

 The mentor set dates for subsequent sessions based on the woman’s gestational age and coordinated with the student care provider. Students were required to report session outcomes or cancellations to the mentor.

####  Continuous care for women in the intervention group

 Each woman received continuous care from a midwifery student from recruitment through six weeks postpartum. The care included nine antenatal visits, attendance at birth from the onset of active labor to two hours postpartum, and four postnatal follow-ups. The care began on the recruitment day; during this session, the student introduced herself, outlined the two-way communication process, and conducted the first care session. All sessions included effective communication, addressing women’s questions, assessing risk factors, and discussing common complaints. The intervention schedule and core components are summarized in Table 1; detailed session-specific activities, the TIDieR checklist, and the intervention fidelity framework are provided in Supplementary Tables S1, S2, and S3, respectively.

**Table 1:** Key components of the Midwifery Student Continuity of Care (MSCOC) intervention

**Phase**	**Timing**	**Delivery mode**	**Key intervention components**
Antenatal continuity of care	26–29 weeks until birth	Two face-to-face visits and seven scheduled telephone/video counselling contacts (nine planned antenatal contacts)	Continuous care by the same student midwife; review of medical records; individualized counselling and communication; childbirth preparation and education; assessment of risk factors; scheduled and responsive support based on women's needs.
Labour and birth	Active labour until 2 h postpartum	Face-to-face	Continuous intrapartum support by the woman's primary student care provider when feasible, from active labour until two hours postpartum; emotional support, information on labour progress, and supportive care during labour.
Postpartum continuity of care	12–24 h postpartum until 6 weeks postpartum	One face-to-face visit and three scheduled telephone/video follow-up contacts	Breastfeeding counselling; maternal and newborn care education; postpartum self-care; mental health counselling; reproductive health and family planning counselling; continued individualized support.

 The supporting students participated in one to two video sessions, but not phone sessions. Both students shared contact numbers with the assigned woman for nonemergency questions between 8 a.m. and 11 p.m., with 24-hour access for emergencies. Initially, women contacted the primary provider; if the primary provider was unavailable, they reached out to the supporting student. The mentor’s phone number was also provided to the women for emergencies.

 The mentor attended all in-person pregnancy sessions, assessed students’ clinical and communication skills, and provided feedback. With the women’s consent, one to two initial phone/video sessions per student were recorded and shared with the mentor for guidance. The mentor was also on-call for the students. To ensure protocol adherence, the students completed a session report form for the mentor after each session.

####  Care during labor and birth for the intervention group

 To ensure continuity of care during labor and birth, women were asked to notify their primary student care provider when labor symptoms began or upon admission for pregnancy termination. The student would then inform the mentor about the woman’s condition. Both remained updated through follow-ups, primarily by phone or occasionally in person, using information provided by the woman or her hospital midwife.

 Once active labor began, the student attended to the woman’s bedside, keeping her informed of labor progress and providing emotional support. The student also assisted with breathing techniques, position changes, and massage. The student remained with the woman until two hours postpartum. If an emergency caesarean section was required, the student’s presence in the operating room had been coordinated, although the staff sometimes restricted it.

####  Routine care for both groups

 All participants had prenatal care at public health centers, with the option to consult private obstetricians or midwives. National standard care for low-risk pregnancies included eight visits during pregnancy and three visits within the first six weeks postpartum. Also, free childbirth preparation classes, led by midwives not involved in ongoing care, were offered at selected centers^27^. In teaching hospitals, midwives assist during labor, and female obstetric residents primarily provide care following a medicalized approach^37^. In other hospitals, midwives work under an obstetrician’s supervision with limited autonomy. Private hospitals offer more respectful care, but continuous midwife support during labor and pharmacological pain relief incur additional costs^28^.

###  Outcomes 

 Among the prespecified primary outcomes, FOC and PPD assessed at 40–50 days postpartum are reported in the current manuscript. Due to space constraints, results of childbirth experience, the third prespecified primary outcome, are reported separately in a companion publication, in which the complete trial protocol is provided as supplementary material.^38^ Secondary outcomes included FOC and PPD assessed at 35–36 weeks of pregnancy and FOC subscale scores at both assessment time points. No hierarchy was prespecified among the primary outcomes, and no multiplicity adjustment was applied; each primary outcome was interpreted separately.

###  Data Collection Tools

 The Wijma Delivery Expectancy/Experience Questionnaire (W-DEQ) was used to assess FOC. It has two versions, each comprising 33 six-point items (scored 0-5) and six sub-scales. W-DEQ-A assesses women’s prenatal expectancies regarding childbirth, while W-DEQ-B evaluates their recent childbirth experiences.^39^ Previous studies have established the validity and reliability of the W-DEQ in the Iranian context.^40^ In the present study, Cronbach’s alpha values were calculated to assess internal consistency and were 0.97 for W-DEQ-A and 0.96 for W-DEQ-B. Alpha values for the subscales ranged from 0.70 to 0.98 for version A and from 0.67 to 0.96 for version B.

 The Edinburgh Postnatal Depression Scale (EPDS), a valid scale to assess depression symptoms during pregnancy and postpartum, was used to assess depression. It consists of 10 four-point Likert items (scored 0-3).^41^ Its validity and reliability have been confirmed in Iran, with a Cronbach’s alpha of 0.77 and an ICC of 0.80 at 6-8 weeks postpartum.^42^ In our study, Cronbach’s alpha was 0.89 at 35-36 weeks of pregnancy and 0.92 at 40-50 days postpartum.

 A demographic and obstetric data form, developed for this study, was used to collect baseline data. Experts confirmed its content validity.

###  Data Analysis

 Data were analyzed using IBM SPSS Statistics for Windows, Version 24.0 (IBM Corp., Armonk, NY, USA). A range check and an independent review of 10% of randomly selected cases were conducted to verify the accuracy of data entry. Questionnaires were reviewed immediately after completion, and participants were asked to complete any missing responses.

 Data analysis was performed after completion of data collection in accordance with the intention-to-treat principle. Separate ANCOVA models were used to compare post-intervention outcomes between the MSCOC and control groups. Baseline outcome values were included as covariates, while intervention group, parity, and hospital type were entered as fixed factors, consistent with the stratified randomization approach. We used complete-case analyses with no imputation for the missing values for the four participants (two per group) at the 35–36-week assessment (secondary outcomes).

 The assumptions of ANCOVA were assessed, including normal distribution of dependent variables and linearity relation between baseline and post-intervention scores at each intervention group, homogeneity of regression slopes, normality of residuals, and homogeneity of variance. All assumptions were considered acceptable for the analyses performed. A two-sided *P* < 0.05 was considered statistically significant.

 Exploratory post-randomization subgroup analyses were conducted based on student attendance during labor and delivery within the MSCOC group; as well as comparison of the MSCOC group without student attendance during labor and delivery with the control group.

## Results

 Recruitment occurred from November 2022 to February 2023, with follow-up completed in July 2023. Of the 60 health centres in Tabriz, 27 met the sampling criteria, and 1-6 women were recruited from each centre. Of the 280 women assessed for eligibility, 93 eligible women were randomly allocated to either the MSCOC group (n = 45) or the control group (n = 48). All randomized women completed postpartum follow-up (primary outcome assessment). However, four participants (two from each group) were not assessed at 35–36 weeks of pregnancy. All randomized participants were analyzed according to the intention-to-treat principle (Figure 1).

**Figure 1:**
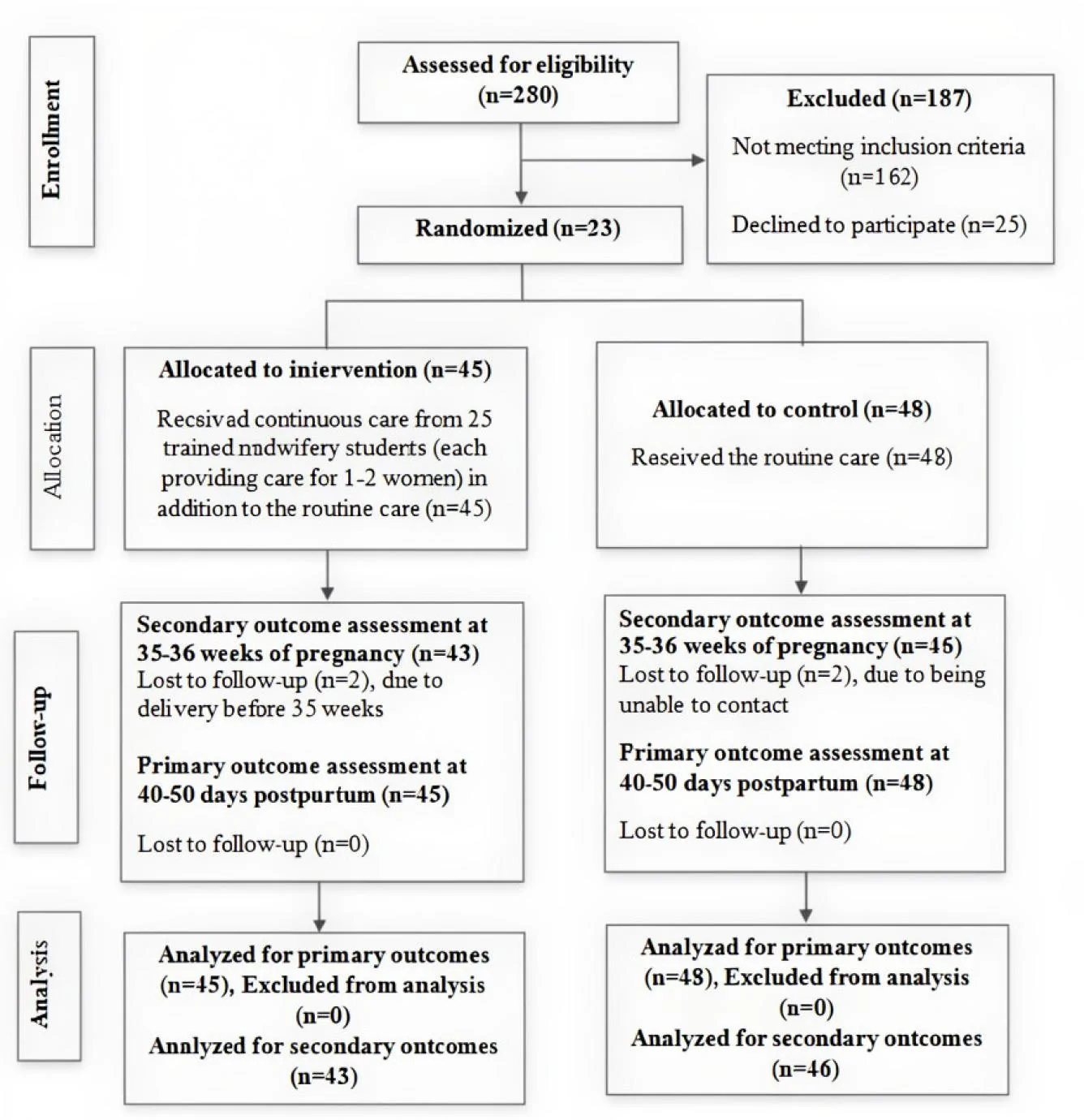
CONSORT Flowchart of Participant Allocation

 Baseline demographic and obstetric characteristics of the participants, including stratification variables (parity and intended hospital type for delivery) by study groups, are presented descriptively in Table 2. The mean age of participants was 26.9 years (SD 4.6); 54% had a diploma or university education, and 50% were primiparous.

**Table 2:** Baseline characteristics of participants by study groups

**Characteristics**	**MSCOC** **(n=45)**	**Control** **(n=48)**
Age (years)	27.1 [6.3]	26.7 [6.6]
Education (Diploma or college)	22 (48.9)	28 (58.3)
Job (Unemployed)	44 (97.8)	41 (85.4)
Adequacy of family income		
Not enough at all	3 (6.7)	3 (6.3)
Relatively enough	38 (84.4)	38 (79.1)
Quite enough	4 (8.9)	7 (14.6)
History of infertility (Yes)	6 (13.3)	2 (4.2)
Planned pregnancy	33 (73.4)	33 (68.8)
Primiparous	22 (46.8)	25 (53.2)
Pregnancy interval from previous birth (years)*	7.7 [3.4]	6.2 [3.2]
Gestational age at recruitment (weeks)	27.5 [1.3]	27.6 [1.2]
Sex of the fetus (Female)	21 (46.7)	23 (47.9)
Satisfaction from previous childbirth*		
Very satisfied or satisfied	11 (47.9)	10 (43.5)
Neither satisfied nor dissatisfied	5 (21.7)	8 (34.8)
Dissatisfied or very dissatisfied	7 (30.4)	5 (21.7)
Intended hospital type for delivery		
Teaching	18 (40)	20 (41.7)
Social security	9 (20)	7 (14.6)
Private/semiprivate	18 (40)	21 (43.7)

MSCOC: Midwifery student continuity of care The data are presented as number (%) or mean [SD] * Among individuals who had a previous birth, n = 23 in each group

 Twenty-eight women (62%) in the MSCOC group and 35 women (73%) in the control group received prenatal care from a private obstetrician, in addition to routine care at the public health centers. In the MSCOC group, 3 women (7%) attended childbirth preparation classes, compared to 7 women (15%) in the control group. During labor and birth, 2 women (7%) in the MSCOC group were accompanied by private midwives and 10 (37%) by family members. In the control group, the corresponding figures were 4 (10%) and 8 (21%). Women attended a mean of 7.3 antenatal sessions (SD = 2.8; range: 1–9) and 2.8 postnatal sessions (SD = 1.3; range: 0–4). Details of the protocol adherence in the intervention group are presented in Table 3.

**Table 3:** Protocol adherence in the intervention group

**Category**	**Reported reason for non-complete adherence**
34 women (76%) attended ≥ 6 antenatal sessions	- Lack of interest (3 cases) - Inadequate follow-up by the student (3 cases) - Spouse’s disapproval (2 cases) - Sudden death of close relatives, opting out of vaginal birth based on obstetrician advice, and preterm birth (each, one case)
29 women (64%) attended ≥ 3 postpartum sessions	
In 21 cases (47%), the student attended during Labor and delivery (In 20 out of 21 cases, the primary student care provider was present)	- Women not informing students (20 cases) due to emergency childbirth, lack of phone access in the delivery room (5 cases), embarrassment to call due to midnight childbirth (2 cases). The 13 other cases that did not provide specific reasons were mostly those who had attended five or fewer antenatal sessions (10 cases). - Inadequate follow-up by the student (4 cases).

 At baseline, the mean total FOC score was numerically higher in the MSCOC group than in the control group (62.1 vs. 54.5). Post-intervention comparisons were performed using ANCOVA models adjusted for baseline FOC score, parity, and hospital type. The MSCOC group had significantly lower mean total FOC scores at 35–36 weeks of pregnancy (31.0 vs. 75.3; adjusted mean difference (AMD) -47.8, 95% CI -54.6 to -40.9) and at 40–50 days postpartum (35.8 vs. 75.8; AMD -43.2, 95% CI -52.7 to -33.7) compared with the control group. (Table 4). At both post-intervention assessments, all FOC subscale scores were lower in the MSCOC group than in the control group (Table S4).

**Table 4:** Comparison of childbirth fear and depression scores between study groups

**Outcomes**	**Baseline**		**35-36 weeks of pregnancy**		**40-50 days postpartum**	
	**MSCOC** **n=45**	**Control** **n=48**	**MSCOC** **n=43**	**Control** **n=46**	**MSCOC** **n=45**	**Control** **n=48**
Total Childbirth fear (0-165)	62.1 (19.9)	54.5 (27.8)	31.0 (18.6)	75.3 (21.1)	35.8 (23.7)	75.8 (26.1)
AMD (95% CI)*	7.0 (-3.0 to 16.9)		-47.8 (-54.6 to -40.9)**		-43.2 (-52.7 to -33.7)**	
Depression (0-30)	11.2 (6.2)	11.2 (6.0)	7.1 (4.2)	13.5 (5.8)	5.3 (4.6)	11.2 (6.6)
AMD (95% CI)*	-0.2 (-2.7 to 2.3)		-6.4 (-8.2 to -4.5)**		-5.9 (-8.1 to -3.7)**	

The data are presented as mean (standard deviation) unless otherwise specified. MSCOC: Midwifery Student Continuity of Care; AMD: adjusted mean difference between the groups; CI: confidence interval The Wijma Delivery Expectancy/Experience Questionnaire (W-DEQ) was used to assess fear of childbirth, and the Edinburgh Postnatal Depression Scale (EPDS) was utilized to assess depression symptoms. Higher scores indicate greater levels of fear or depression. * ANCOVA was used, adjusted for parity and hospital type for the baseline comparisons, and adjusted for parity, hospital type, and baseline values for post-intervention comparisons **P < 0.001

 The mean depression score at baseline was 11.2 in both the intervention and control groups. However, significantly lower scores were observed in the intervention group at both postintervention follow-ups: gestational weeks 35-36 (7.1 vs. 13.5; AMD -6.4; 95% CI -8.2 to -4.5) and 40-50 days postpartum (5.3 vs. 11.2; AMD: -5.9; 95% CI: -8.1 to -3.7).

 There were no statistically significant differences between subgroups of the MSCOC group with and without student attendance during labor and delivery in terms of frequency of primiparity, main antenatal care provider, or type of delivery. However, the subgroup with student attendance had significantly higher frequency of attendance at six or more antenatal sessions (95% vs 58%, *P* = 0.005) and at three or more postpartum sessions (95% vs 38%, *P* < 0.001). They also had a higher frequency of delivering at a teaching hospital (52% vs 29%, *P* = 0.03) (Table S5).

 As an exploratory analysis, we examined outcomes according to student attendance during labor and delivery. At 35-36 weeks of pregnancy, no statistically significant differences were observed between the two subgroups of the MSCOC group, with and without student attendance during labor and delivery, in the mean total FOC, FOC subscales, or the depression scores. However, at 40–50 days postpartum, participants with student attendance had lower loneliness and fear subscale scores. Total FOC and depression scores were also numerically lower (by 12 and 2.5 points, respectively), but the differences were not statistically significant (Table S6).

 At 40-50 days postpartum, no statistically significant differences were observed between the subgroup without student attendance during labor and delivery and the control group in the mean scores for total FOC and PPD scores. The group without student attendance had significantly lower mean scores than the control group on two FOC subscales: the lack of self-efficacy and the concerns for the child (S3).

## Discussion

 In this study, we introduced the MSCOC model in Iran, a novel approach in a context where MCOC has not been widely implemented. The MSCOC group showed lower post-intervention FOC severity and PPD symptoms than the control group. The observed reduction in W-DEQ scores exceeded commonly used thresholds for clinically relevant fear of childbirth, suggesting that the intervention effect may have clinical relevance in addition to statistical significance. Moreover, the reduction in EPDS scores moved participants toward a lower-risk range based on established screening thresholds for postpartum depressive symptoms.

 The findings are consistent with previous Swedish research highlighting the benefits of MCOC for women experiencing FOC.^43-45^ Another qualitative study reported that women experienced reduced stress and increased autonomy through MCOC.^45^ Additionally, an experimental study indicated that MCOC offers valuable opportunities for decision-making participation, and a sense of control for women with FOC.^44^ The establishment of a mutually trusting relationship between women and midwives can reduce their fear.^46^ A prior Iranian study found that the childbirth preparation courses approved by the Ministry ofHealth did not significantlyreduce FOC.^47^ While these courses enhance knowledge, their effectiveness in promoting a positive childbirth experience is limited.^48^ This is likely due to thelack of individualized, continuity-based care and insufficient focus on FOC.

 Although we found no studies examining the effect of MSCOC on FOC, existing literature suggests that MSCOC shares several characteristics with MCOC that may help alleviate FOC. This model has been shown to enhance women’s empowerment through supportive care, strengthening the partnership between women and students, and providing evidence-based care.^19-21^ Qualitative studies indicated thatwomen perceive the student midwife’s attendance in labor and delivery as essential.^22,23^ They view the student midwife as their advocate, helping them express their needs, expectations, and concerns, fostering calm and security, and enabling greater focus on the childbirth process.^19^ Our exploratory subgroup analysis showed that participants with student attendance during labor had lower FOC subscale scores related to fear and loneliness; however, these findings represent associations and should not be interpreted as causal effects of student attendance. Students were more likely to attend labor for women who had participated in most prenatal and postpartum sessions, consistent with findings from a Swedish study.^44^ This pattern may reflect stronger engagement and relationship-building through repeated interactions, although differences in motivation or adherence may also have contributed. Since student attendance during labor was not randomized, these findings should be interpreted as exploratory associations rather than causal effects. Notably, despite the generally negative childbirth experiences reported in teaching hospitals in our context^49-51^, a higher proportion of women in the MSCOC group gave birth in teaching hospitals. The availability of a familiar student midwife and continuous support may have contributed to women’s willingness to give birth in these settings; however, differences in hospital type between subgroups may also have influenced the observed findings. Therefore, these subgroup results should be considered hypothesis-generating and interpreted cautiously.

 While previous studies did not directly address the effect of MSCOC on PPD, our findings align with research suggesting that psychosocial interventions based on continuous care can significantly reduce PPD risk.^52,53^ Our results are consistent with a Thai study, which found that midwife and family support reduced PPD symptoms.^54^ Studies indicate that brief interventions, such as digital content orlimited midwife contacts, donot significantly affect PPD,^55,56^ but MCOC enhances midwives’ role in perinatal mental health by providing sufficient time and frequent interactions in a supportive environment.^8^ However, midwives face challenges due to insufficient training in perinatal mental health.^57^ Specialized training in relevant psychosocial skillsimproves midwives’ competence and confidence, positively impacting perinatal mental health, especially in low- and middle-income countries.^8^ In thisstudy,MSCOC providers received such training from a mentor withexpertise in perinatal mental health and midwifery-led counseling, which likely contributed to the observed positive effects.

 The study also highlights the challenges related to student attendance during labor and delivery, with only 50% attendance compared to 93% in Australia.^20^ This discrepancy can be attributed to the voluntary nature of student participation in this study, limited logistical support, and the lack of mandatory educational requirements. The Australian Nursing and Midwifery Council now mandates student involvement during labor as part of the MSCOC requirements.^20^ However, we observed that students were more likely to attend labor for women who had participated in most prenatal sessions, which aligns with a Swedish study.^44^ This reinforces the importance of building strong, trusting relationships between midwives and women.^15,58^

 In this trial, strict adherence to random sequence generation and central allocation concealment minimized selection bias. Although blinding of participants, student care providers, and outcome assessors was not feasible, efforts were made to reduce potential performance and detection bias by withholding study outcomes from participants and care providers and emphasizing accurate questionnaire completion. The minimal loss to follow-up reduced the risk of attrition bias. Active engagement between the mentor and students likely bolstered adherence to the intervention. Moreover, enrolling participants from all eligible urban health centers in the capital city may have enhanced the generalizability of our findings. Given that most maternity indices in this city (the center of Iran’s fifth-largest province) align with the mean urban indices, our findings may be relevant to similar urban settings across Iran.

 The study also highlighted thefeasibilityofintegrating MSCOC within our midwifery educationsystem;However, several limitations should be considered.

 The intervention was supervised by a single qualified mentor who guided 25 students. As this project was conducted as part of her doctoral thesis, her high motivation and commitment may not be fully representative of larger-scale implementation involving multiple mentors. Due to the mentor’s workload and participants’ limited internet access, a virtual group could not be established, and some intervention sessions were conducted by telephone. We did not systematically record the frequency of phone calls or text messages exchanged between women and student midwives; therefore, the contribution of these unscheduled supportive interactions to the overall intervention effect remains unclear.

 Due to the nature of the intervention, blinding of participants, student care providers, and the mentor who also collected the data was not feasible. Because both primary outcomes were self-reported by participants, the possibility of performance and detection bias cannot be completely excluded.

 Although the intervention was delivered by multiple students, clustering by student was not accounted for in the analyses because each student provided care to only one or two women as the primary care provider. Therefore, some residual provider-level variability cannot be completely excluded. We conducted no multiplicity adjustment due to the three pre-specified primary outcomes, which could lead to increasing type I error. However, because in all primary outcome comparisons P-values were less than 0.001, with Bonferroni correction, the errors will be less than 0.003.

 Potential co-interventions may also have influenced maternal outcomes. Although private obstetrician care, childbirth preparation classes, and labor companionship were documented, residual confounding from these factors cannot be completely excluded. Future studies with larger sample sizes should further evaluate their potential influence.

 The broad exclusion criteria, smartphone/internet requirements for intervention delivery, and recruitment from urban health centers may limit the generalizability of findings to rural populations, women with limited digital access, and higher-risk pregnancies. Additionally, the limited representation of women from higher socioeconomic backgrounds may restrict applicability to more diverse populations.

 The lack of financial incentives or educational requirements for student participation, along with insufficient support for project implementation at hospitals, may have affected student motivation and adherence.

 Baseline differences in FOC scores between groups may have influenced the observed changes. Although baseline-adjusted ANCOVA models were used to account for initial differences, residual confounding and regression to the mean cannot be completely excluded.

 As PPD and FOC can have negative long-term effects on maternalphysical and psychologicalhealth, infants’ emotional and cognitive development,^7^ and family well-being,^59^ the results of this study offer promising potential for improving family health outcomes. The program offers dual benefits by providing mothers with personalized, woman-centered care while enabling student midwives to enhance their midwifery and interpersonal skills, boost confidence, and improve competence through practical experience.^17^ However, there are ambiguities regarding the pedagogical intent and the definition and measurement of learning outcomes for the MSCOC.^17^ Given these ambiguities and our study’s limitations, we recommend further research across various academic centers. Such research should include action research to explore MSCOC’s benefits, challenges, and implementation strategies. It seems that incorporating MSCOC into student curricula, mandating mentoring for academic midwives, involving clinical midwives for student support, and providing facilities could enhance MSCOC implementation and outcomes.

## Conclusion

 Our findings highlight the potential for integrating MSCOC into the midwifery education system in Iran. The study suggests that MSCOC reduces fear of childbirth and postpartum depression symptoms in women. These findings could motivate further research to explore the advantages, challenges, and potential national adoption of the program, particularly in low- and middle-income countries.

## Competing Interests

 The authors declare that they have no competing interests.

## Consent for Publication

 Not applicable.

## Data Availability Statement

 The corresponding author will make deidentified participant datasets available for research purposes to researchers affiliated with academic institutions and others upon reasonable request immediately after the publication of the results.

## Ethical Approval

 This study received scientific approval (Grant no: 69665) and ethical approval (Ethical code: IR.TBZMED.REC.1401.094) from Tabriz University of Medical Sciences, Tabriz, Iran.

 All participants provided written informed consent before enrolling in the study. The study was conducted in accordance with relevant guidelines and regulations.

## Supplementary files

Supplementary file 1 contains Table S1-S6.
