# Supplementary material for: Effect of Midwifery Students’ Continuity of Care on Childbirth Fear and Postpartum Depression: An Individually Randomized Two-Arm Superiority Trial
Source: Health Promot Perspect. 2026 Aug 21;16(2):217–26. doi: 10.34172/hpp.45154 (PMC13723428; doi:10.34172/hpp.45154)
Supplement: Supplementary file 1 [file hpp-16-217-s001.pdf]

Jafari et al, Health Promotion Perspectives 2026;16(2):217-S1
doi:10.34172/hpp.45154
https://hpp.tbzmed.ac.ir

Supplementary Table 1. Detailed session-by-session content of the Midwifery Student Continuity of Care (MSCOC) intervention

No.	Method	Content
Pregnancy sessions		
1	In-person care (weeks 26-29)	Review of medical records, lab tests, and sonography, as well as clinical examination. Counselling on nutrition, exercise, breathing, and relaxation techniques.
2	Four phone/video counselling sessions spaced 7-10 days apart	The stages of normal vaginal birth and the relevant care for each stage.
3		Childbirth fear, its consequences, and management strategies.
4		labor pain, influencing factors, and pain relief methods (both pharmacological and non-pharmacological).
5		Reviewing previous sessions and discussing relaxation methods and breathing techniques for labor and birth.
6	In-person care (weeks 35-36)	Conduct a clinical examination, and practice relaxation and breathing techniques.

7	Weekly phone/video counselling sessions from 37 weeks of pregnancy until delivery	Preparation for childbirth, including supplies, hospital arrangements, transportation, care for other children, labor symptoms, and emergency caesarean preparation.
8		Newborn care, including skin-to-skin contact, delayed cord clamping, and breastfeeding initiation.
9 & 10		Review prior sessions based on individual needs.
Postpartum sessions		
1	In-person care at the hospital<br>(12-24 h postpartum)	Breastfeeding, neonatal care, personal hygiene, and perineal care.
2	Phone/video counselling<br>(3-5 d postpartum)	Breastfeeding, maternal nutrition, and postpartum blues.
3	Phone/video counselling<br>(7-10 d postpartum)	Breastfeeding continuation, postpartum depression, resumption of sexual intercourse, and family planning.
4	Phone/video counselling<br>(20-30 d postpartum)	Conclude continuous care, appreciate women's collaboration, and ensure responsiveness to their questions up to six weeks postpartum.

Supplementary Table S4. TIDieR checklist for the Midwifery Student Continuity of Care (MSCOC) intervention

TIDieR item	Description	Location
**1. Brief name**	Midwifery Student Continuity of Care (MSCOC)	Title; Methods—Intervention
**2. Why**	To provide continuity of care through an ongoing student–woman relationship, individualized emotional support, and childbirth preparation.	Introduction; Methods—Intervention
**3. Materials**	National maternal health guidelines, educational PDFs, audio/video materials, session guidance, and session report forms.	Methods—Student training and mentoring; intervention
**4. Procedures**	Final-year midwifery students provided continuity of care across the antenatal, intrapartum, and postpartum periods according to a predefined protocol, including scheduled contacts, individualized counselling, risk assessment, childbirth preparation, labour support, postpartum follow-up, and responsive communication.	Methods—Intervention; Table 1; Supplementary table S1
**5. Providers**	Twenty-five final-year midwifery students delivered care under the supervision of an experienced midwifery instructor who mentored the students throughout the trial.	Methods—Participants; Intervention
**6. Mode of delivery**	Face-to-face visits, telephone/video counselling, online communication, and in-person labour support when feasible.	Methods—Intervention; Table 1; Supplementary table S1
**7. Location**	Public health centres, participating hospitals, and remote communication platforms in Tabriz, Iran.	Methods—Recruitment and Intervention
**8. When and how much**	Care was provided from 26–29 weeks of gestation until six weeks postpartum, including two face-to-face visits and seven scheduled telephone/video counselling contacts (nine planned antenatal contacts) in pregnancy, labour support from active	Methods—Intervention; Table 1; Supplementary table S1

	labour to two hours postpartum when feasible, and four postpartum follow-ups.	
**9. Tailoring**	Core intervention components were standardized; counselling topics and supportive discussions were adapted according to women's needs, concerns, and clinical circumstances.	Methods—Intervention
**10. Modifications**	No planned modifications to the intervention were made after trial initiation.	Methods—Intervention; Discussion
**11. Fidelity planned**	Fidelity was supported through student training, mentor supervision, mentor feedback, selected session recordings, on-call support, and session reports.	Methods—Student training and mentoring; intervention
**12. Fidelity actual**	Fidelity was assessed through completion of antenatal and postpartum contacts and student attendance during labour and birth. In the intervention group, 76% attended ≥6 antenatal sessions, 64% attended ≥3 postpartum sessions, and 47% received student attendance during labour and birth. Reasons for incomplete adherence are reported in Table 3.	Results; Table 3

Supplementary Table S3 Intervention Fidelity Framework

Intervention component	Mandatory	Flexible	Fidelity monitoring
Student training workshops	Four prespecified workshops before participant recruitment	–	Workshop attendance
Mentor supervision	Ongoing supervision, feedback, and on-call support throughout the trial	Additional guidance according to students' needs	Session reports, mentor feedback, and selected recorded sessions
Scheduled antenatal contacts	Nine planned antenatal contacts delivered according to the protocol	Timing could be adjusted according to gestational age and participants' availability	Session report forms
Communication mode	Face-to-face visits plus scheduled telephone/video counselling	Telephone or video format according to feasibility	Session report forms
Childbirth counselling	Core counselling topics delivered according to the intervention protocol	Discussions tailored to individual women's needs and concerns	Mentor review and session reports
Labour and birth support	Student attendance from active labour until two hours postpartum was intended	Depended on notification by the woman, labour timing, student accessibility, and hospital policies	Attendance records
Postpartum follow-up	Four planned postpartum contacts	Timing adapted when required	Session report forms
Additional participant support	Access to student care providers (8 a.m.–11 p.m. for non-emergency questions and 24-hour emergency contact) throughout the intervention	Frequency and timing of woman-initiated contacts	Not systematically quantified

Table S4. Comparison of childbirth fear subscale scores between the study groups

Subscale	Baseline		35-36 weeks			40-50 days postpartum		
	**MSCOC n=45**	**Control n=48**	**MSCOC n=43**	**Control n=46**	Adjusted mean differences (95% CI)	**MSCOC n=45**	**Control n=48**	Adjusted mean differences (95% CI)
Lack of self-efficacy (0-50)	15.4 (8.7)	13.0 (9.0)	7.1 (6.0)	17.2 (7.2)	-11.1 (-13.5 to -8.7)	7.5 (7.8)	20.0 (8.8)	-13.6 (-16.7 to -10.5)
Lack of positive anticipation (0-20)	3.1 (2.5)	3.0 (2.6)	1.7 (2.1)	5.0 (2.1)	-3.4 (-4.2 to -2.5)	3.5 (4.7)	7.0 (3.8)	-3.5 (-5.3 to -1.8)
Loneliness (0-40)	19.6 (7.2)	16.6 (9.8)	7.7 (7.1)	22.7 (7.2)	-16.0 (-18.7 to -13.3)	8.4 (8.1)	19.4 (9.7)	-12.0 (-15.6 to -8.4)
Fear (0-25)	16.6 (4.4)	14.7 (6.1)	11.0 (4.5)	17.8 (4.2)	-7.4 (-9.0 to -5.7)	12.4 (4.5)	18.4 (4.6)	-6.4 (-8.3 to -4.6)
Concerns for the child (0-10)	2.3 (2.8)	2.9 (3.2)	0.7 (1.5)	5.3 (3.3)	-4.3 (-5.4 to -3.2)	0.7 (1.5)	3.7 (3.1)	-3.0 (-4.0 to -1.9)
Concerns about losing control (0-15)	5.1 (2.8)	4.3 (3.4)	2.6 (2.8)	7.3 (2.8)	-5.0 (-6.1 to -4.0)	3.3 (3.2)	7.3 (3.0)	-4.1 (-5.3 to -2.9)

Data are presented as means (standard deviations) unless otherwise specified. Adjusted mean differences were calculated as MSCOC minus control using ANCOVA models adjusted for baseline values, parity, and hospital type; therefore, negative values indicate lower fear of childbirth scores in the MSCOC group. MSCOC, Midwifery Student Continuity of Care; W-DEQ, Wijma Delivery Expectancy/Experience Questionnaire; higher scores indicate greater fear of childbirth across all subscales. P < 0.001 for all post-intervention comparisons.

Table S5. Exploratory post-randomization subgroup comparison of obstetric characteristics according to midwifery student attendance during labor and delivery in the MSCOC group

Variable	Midwifery student attendance: Yes (n=21)	Midwifery student attendance: No (n=24)	P value
Primiparous	10 (48)	12 (50)	1.0*
Main antenatal care provider (Midwife)	9 (43)	8 (33)	0.55*
Attended >=6 pregnancy sessions	20 (95)	14 (58)	0.005**
Attended >=3 postpartum sessions	20 (95)	9 (38)	<0.001**
Type of delivery			0.88**
Non-instrumental vaginal delivery	15 (71)	18 (75)	
Emergency caesarean section	5 (24)	4 (17)	
Elective caesarean section	1 (5)	2 (8)	
Place of delivery			0.03**
Teaching hospital	11 (52)	7 (29)	
Social Security hospital	6 (29)	3 (13)	
Private hospital	4 (19)	14 (58)	

Data are presented as number (%) unless otherwise specified. *Chi-squared test. ** Fisher's exact test

Table S6. Exploratory post-randomization subgroup analysis of childbirth fear and depression scores according to midwifery student attendance during labor and delivery

**Outcome**	**35-36 weeks of pregnancy**			**40-50 days postpartum**			Adjusted mean differences (95% CI) at **40-50 days postpartum** *	
	Midwifery student attendance during labor and delivery in the MSCOC group		**Control n=46**	Midwifery student attendance during labor and delivery in the MSCOC group		**Control n=48**	**G1 vs G2**	**G2 vs control**
	**Yes (G1) n=21**	**No (G2) n=22**		**Yes (G1) n=21**	**No (G2) n=24**			
Childbirth fear: Total (0-165)	30.4 (18.6)	30.7 (19.0)	75.3 (21.1)	28.6 (21.0)	42.2 (24.6)	75.8 (26.1)	-12.1 (-26.0 to 1.9)	-10.5 (-27.8 to 7.0)
Lack of self-efficacy (0-50)	7.4 (6.0)	7.0 (6.0)	17.2 (7.2)	6.1 (8.0)	8.8 (7.5)	20.0 (8.8)	-7.0 (-7.0 to 0.1)	-6.4 (-11.4 to -1.3)**
Lack of positive anticipation (0-20)	2.2 (2.6)	1.2 (1.3)	5.0 (2.1)	3.6 (4.5)	3.5 (5.0)	7.0 (3.8)	0.3 (-3.0 to 3.6)	-2.8 (-5.8 to 0.3)
Loneliness (0-40)	7.5 (6.9)	7.9 (7.4)	22.7 (7.2)	5.4 (6.2)	11.0 (8.7)	19.4 (9.7)	-5.4 (-10.8 to -0.1)**	-0.8 (-7.0 to 5.5)
Fear (0-25)	10.2 (3.8)	12.0 (5.0)	17.8 (4.2)	10.5 (4.6)	14.1 (3.8)	18.4 (4.6)	-2.6 (-5.2 to -0.1)**	-1.5 (-3.9 to 0.9)
Concerns for the child (0-10)	1.1 (1.9)	0.4 (1.0)	5.3 (3.3)	0.7 (1.6)	0.8 (1.5)	3.7 (3.1)	0.0 (-1.1 to 1.2)	-2.6 (-4.4 to -0.8)**
Concerns about losing control (0-15)	2.0 (2.6)	3.1 (2.9)	7.3 (2.8)	2.3 (3.0)	4.1 (3.3)	7.3 (3.0)	-1.1 (-3.4 to 1.1)	-1.8 (-3.6 to 0.0)
Depression (0-30)	7.5 (4.6)	7.0 (3.8)	13.5 (5.8)	3.7 (3.0)	6.8 (5.4)	11.2 (6.6)	-2.5 (-5.4 to 0.4)	-1.2 (-4.5 to 2.2)

Data are presented as means (standard deviations) unless otherwise specified. MSCOC: Midwifery Student Continuity of Care; AMD: adjusted mean difference; CI: confidence interval; G1: participants in the MSCOC group with midwifery student attendance during labor and delivery; G2: participants in the MSCOC group without midwifery student attendance during labor and delivery. The Wijma Delivery Expectancy/Experience Questionnaire (W-DEQ) was used to assess fear of childbirth, and the Edinburgh Postnatal Depression Scale (EPDS) was used to assess depressive symptoms; higher scores indicate greater levels of fear or depression. *After excluding the four participants with missing 35–36-week assessments (two from G2 and two from the control group), complete-case ANCOVA models were used, adjusted for 35–36-week values, parity, and hospital type. Statistical significance was inferred when the 95% CI of the AMD did not include zero. **$P < 0.05$
